# A comparative study of transscleral sutured intraocular lens fixation and sutureless flanged intraocular lens fixation

**DOI:** 10.1186/s12886-023-02782-y

**Published:** 2023-01-17

**Authors:** Ying Cui, Qiyan Li, Xiangyu Shi, Dan Zhou

**Affiliations:** Beijing Tongren Eye Center, Beijing Tongren Hospital, Capital Medical University, Beijing, 100730 China

**Keywords:** Intraocular lens, Sutureless flanged fixation, Optical coherence tomography, Higher-order aberrations

## Abstract

**Background:**

To compare the intraocular lens (IOL) tilt and decentration and visual outcomes of transscleral sutured IOL fixation and sutureless flanged IOL fixation. To investigate the influence of IOL tilt and decentration on internal higher-order aberrations (HOAs) in these two techniques.

**Methods:**

Patients who received transscleral sutured or sutureless flanged IOL fixation procedures were included in this prospective, non-randomized, comparative study. Corrected distance visual acuity (CDVA) was measured at baseline and at every postoperative visit for 12 months. IOL tilt and decentration were measured using a second-generation anterior segment optical coherence tomography (Casia2) and internal HOAs were measured using iTrace Visual Function Analyzer at 3 months postoperatively.

**Results:**

The study included 27 eyes from the transscleral sutured IOL fixation group and 26 eyes from the sutureless flanged IOL fixation group. There was no significant difference in CDVA between the two groups at all time points. The two groups did not differ in refractive difference from the predicted value, corneal endothelial cell loss, IOL tilt, IOL decentration, internal astigmatism or internal HOAs. Vertical IOL decentration significantly correlated with total internal optical aberration (*r* = 0.288, *P* = 0.036), total internal HOA (*r* = 0.440, *P* = 0.001), internal coma (*r* = 0.348, *P* = 0.001), vertical internal coma (*r* = 0.388, *P* = 0.004), average height of modulation transfer function (*r* = − 0.364, *P* = 0.007) and Strehl ratio (*r* = − 0.297, *P* = 0.031). Horizontal IOL decentration significantly correlated with horizontal internal coma (*r* = 0.312, *P* = 0.023).

**Conclusions:**

Transscleral sutured IOL fixation and sutureless flanged IOL fixation had similar IOL positions and visual outcomes. IOL decentrations correlated with internal HOAs and thus should be avoided.

## Background

In patients with insufficient capsular support, intraocular lens (IOL) can be implanted in the anterior chamber, fixed on the iris or fixed in the ciliary sulcus with transscleral sutures [1]. Since anterior chamber IOL can accelerate corneal endothelial cell loss and iris fixed IOL can cause recurrent iritis, transscleral sutured IOL fixation technique was preferred by many surgeons. However, suture-related complications remained a major concern, such as suture exposure and subluxation of the IOL after suture breakage. In a 6-year study involving 61 eyes, suture breakage was observed in 26.2% of cases after scleral fixation of IOLs using 10–0 polypropylene sutures [2].

In 2007 and 2008, Gabor and Agarwal reported the technique of sutureless intrascleral IOL fixation, which was modified by others in later studies [3–7]. The basic principle of this technique is incarceration of the IOL haptics in a scleral tunnel parallel to the limbus. In 2017, Yamane et al. developed a technique named “flanged IOL fixation” [8]. This procedure does not require peritomy or scleral flap dissection. The IOL can be fixed firmly to the sclera with the enlarged tips of the haptics.

Previous studies have compared the IOL positions and the visual outcomes between sutured transscleral fixation and sutureless intrascelral fixation techniques, but there are limitations [9–12]. First, most of these studies used ultrasound biomicroscopy (UBM) or Scheimpflug photography to measure IOL tilt and decentration, which had relatively low accuracy and reproducibility due to alignment issues or image quality. Second, the astigmatism measured by optometry comes from both the cornea and the IOL. The influence of IOL malposition on internal astigmatism could not be decided accurately. Third, higher-order aberrations (HOAs) was not included in the evaluation of visual outcomes. In this study, we aimed to compare the two IOL fixation techniques in a more detailed manner, using a second generation swept-source anterior segment optical coherence tomography (AS-OCT) to measure the IOL tilt and decentration and using the iTrace Visual Function Analyzer to measure internal astigmatism and HOAs.

## Methods

### Study design and patients

This prospective comparative study was carried out at Beijing Tongren Eye Center, Beijing Tongren Hospital from June 2020 to June 2021. All of the patients aged 18+ years who received transscleral sutured or sutureless flanged IOL fixation procedures by a single surgeon were included. The diagnosis of the participants included crystalline lens dislocation, IOL dislocation and aphackic eyes. Exclusion criteria included severe retinal diseases (retinal detachment, proliferative diabetic retinopathy, retinal vein occlusion and age-related macular degeneration), uncontrolled glaucoma, corneal endothelial cell density less than 1000/mm2 and inflammation of the sclera. The study protocol was approved by the ethics committee of Beijing Tongren Hospital (approval ID: TRECKY2021–061) and adhered to the guidelines of the Declaration of Helsinki. Informed consent was obtained from all the participants.

### Surgical procedures

A Constellation Vision System (Alcon Laboratories, Inc.) was used to perform complete 23G vitrectomy before IOL implantation.

#### Transscleral sutured IOL fixation

After partial limbal peritomy, two lamellar sclerotomy of 1/3 scleral thickness were made 2 mm from the limbus at 3:00 and 9:00 and were dissected outward (away from the limbus) to create two “scleral pockets”. A 29G needle was used to penetrate the bed of one scleral pocket 2.2 mm posterior to the limbus. A straight needle attached to a 10–0 polypropylene suture was used to penetrate the bed of the opposite scleral pocket 2.2 mm posterior to the limbus. The straight needle was inserted into the lumen of the 29G needle and externalized onto the sclera, leaving the suture extending across the posterior chamber. The suture was pulled outside from the corneal incision, divided and tied to the opposite eyelets of a 4-haptic IOL (Akreos Adapt-AO, Bausch & Lomb). The IOL was folded and inserted into the posterior chamber. The IOL was sutured onto the scleral bed and the knots were buried under the scleral pockets.

#### Sutureless flanged IOL fixation

**Fig. 1:**
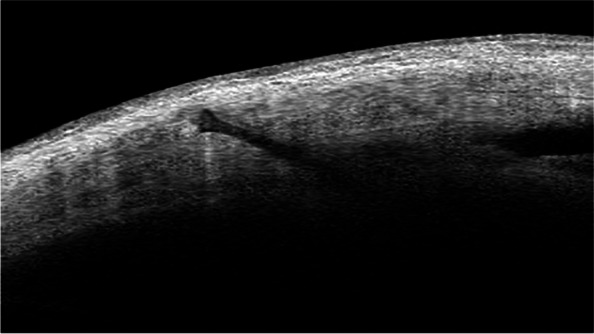
Optical coherence tomography (OCT) image showing the flange of the IOL haptic is fixed in the sclera

Flanged IOL fixation was introduced by Yamane et al. in 2017 [8]. We used 29G needles instead of the 30G thin-wall needles used by Yamane. A 3-piece IOL (Tecnis ZA9003, Abbott Medical Optics) was inserted into the anterior chamber using an injector. An angled sclerotomy was made through the conjunctiva using a 29G needle 2 mm from the limbus. The leading haptic was inserted into the lumen of the needle with a 23G forceps. A second sclerotomy was made 180°from the first one. The trailing haptic was inserted into the lumen of the second needle. Both haptics were externalized onto the conjunctiva using the double-needle technique. The ends of the haptics were cauterized to make a flange. The haptics were pushed back and docked into the scleral tunnels (Fig. 1).

### Ophthalmic examinations

All patients underwent ophthalmic examinations including measurement of uncorrected distance visual acuity (UDVA), corrected distance visual acuity (CDVA), intraocular pressure (IOP), slit-lamp examination, subjective refraction and fundus examination at baseline and at every postoperative visit for at least 12 months.

### Measurement of IOL tilt and decentration using AS-OCT

**Fig. 2:**
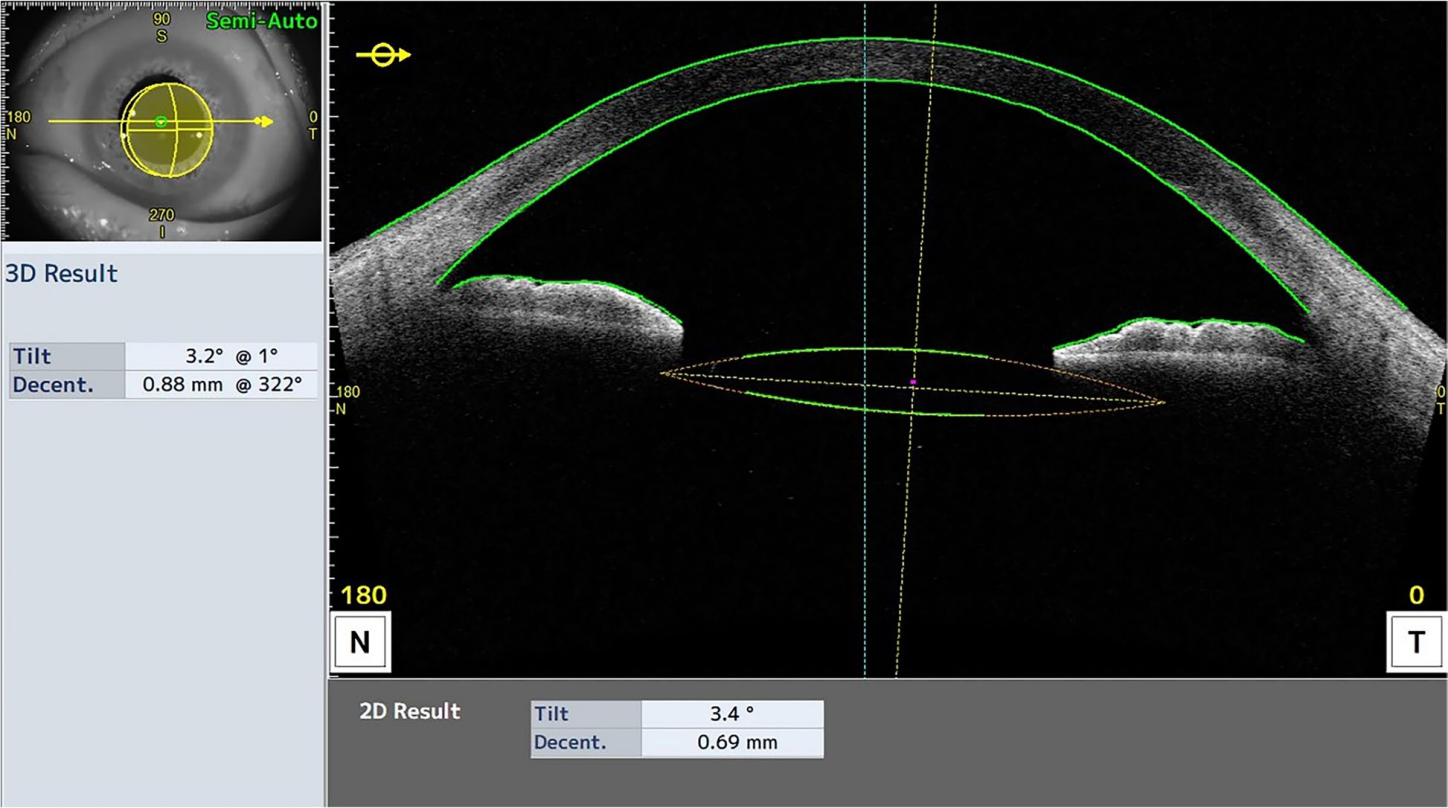
Measurement of intraocular lens (IOL) tilt and decentration using a swept-source optical coherence tomography (Casia2). The optic axis of the IOL (yellow line) and the corneal topographic axis (blue line) are automatically drawn and the IOL tilt and decentration are automatically calculated with the built-in software

IOL tilt and decentration were measured 3 months postoperatively using a swept-source AS-OCT (Casia2; Tomey Corporation). The examination was performed after mydriasis. The Casia2 uses a 1310 nm swept-source laser at a frequency of 0.3 seconds. In the IOL scan measurement mode, it generates 4 distinct AS-OCT images from 4 different angles (0 to 180, 90 to 270, 45 to 225 and 135 to 315 degree) and a 3-dimensional (3D) analysis of the results [13]. It automatically measures the tilt and decentration of the IOL relative to the corneal topographic axis [14]. If the anterior or posterior surface of the IOL failed to be detected correctly, the semi-automatic mode was used and the surfaces of the IOL were delineated manually. Horizontal (0 to 180 degree) and vertical (90 to 270 degree) images were used to analyze the IOL tilt and decentration. An example image is shown in Fig. 2.

### Measurement of internal astigmatism and HOAs

The iTrace Visual Function Analyzer (iTrace, Tracey Technologies Corporation) was used to measure internal astigmatism and HOAs 3 months postoperatively. Patients were examined with undilated pupil and stayed in dark environment for 10 minutes before examination. Firstly, the placido-disc-based corneal topography was obtained, giving the corneal aberration. Secondly, the ray-tracing-principle-based wave front was measured, giving the total optical aberration. The internal optical aberration was then calculated atomically by subtracting corneal aberration from the total optical aberration.

### Statistical analysis

Statistical analysis was conducted with SPSS for Windows (v. 20.0, SPSS Inc.). Numerical variables were presented as mean ± standard deviation and Student’s t test was used to compare differences between groups. Categorical variables were described by frequencies and percentages and tested by χ2 test. Spearman correlation analysis was used to investigate the relationship between IOL tilt and decentration and internal astigmatism and HOAs. Two-sided *P* < 0.05 was considered as statistically significant in all analyses.

## Results

The study consisted of 27 eyes (24 patients) from the transscleral sutured IOL fixation group and 26 eyes (26 patients) from the sutureless flanged IOL fixation group. The mean ages were 56.5 ± 10.1 years (range, 36–71 years) for the sutured group and 56.5 ± 13.9 years (range, 28–82 years) for the flanged IOL fixation group. The two groups did not differ significantly in age, sex, UDVA, CDVA, IOP, axial length or corneal endothelial cell density at baseline. There were 22 dislocated crystalline lenses, 22 dislocated posterior chamber IOLs and 9 aphakic eyes. The two groups did not differ significantly in the distributions of preoperative diagnosis (*P* = 0.461). The patient characteristics are shown in Table 1.

**Table 1:** Patient demographics and clinical characteristics at baseline

Parameters	Sutured fixation (***n*** = 27)	Flanged fixation (***n*** = 26)	***P***
Age (mean ± SD, years)	56.5 ± 10.1	56.5 ± 13.9	0.995
Sex (male/female)	18/9	18/8	0.842
UDVA (mean ± SD, logMAR)	1.30 ± 0.58	1.32 ± 0.44	0.881
CDVA (mean ± SD, logMAR)	0.34 ± 0.30	0.39 ± 0.44	0.607
AL (mean ± SD, mm)	24.07 ± 1.69	24.67 ± 1.45	0.212
IOP (mean ± SD, mmHg)	16.7 ± 5.4	17.5 ± 5.7	0.605
Endothelial cell density (mean ± SD, /mm2)	2277 ± 722	1972 ± 689	0.157
Preoperative diagnosis (crystalline lens dislocation/IOL dislocation/aphakic eyes)	13/9/5	9/13/4	0.461

*AL*, axial length, *CDVA* corrected distance visual acuity, *IOP* intraocular pressure, *UDVA* uncorrected distance visual acuity

The CDVA in both groups improved significantly at 1, 3, 6 and 12 months postoperatively compared with baseline (*P* = 0.002, *P* < 0.001, *P* < 0.001 and *P* < 0.001 for sutured group, and *P* = 0.011, *P* = 0.005, *P* = 0.002 and *P* = 0.002 for sutureless flanged fixation group, respectively). However, there was no difference in CDVA between the two groups at all time points (*P* > 0.05).

There was no significant difference in astigmatism measured by subjective refraction between the two groups at 3 months postoperatively (*P* = 0.272). Refractive difference was defined as the difference between the spherical equivalent at 3 months postoperatively and the intended refractive error. Corneal endothelial cell loss was defined as the difference between the endothelial cell density 3 months postoperatively and at baseline. There was no significant difference between the two groups in refractive difference (*P* = 0.618) and corneal endothelial cell loss (*P* = 0.250) (Table 2).

**Table 2:** Comparison of visual acuity, astigmatism, refractive difference and endothelial cell loss between the 2 groups at 3 months postoperatively

Parameters	Sutured fixation (***n*** = 27)	Flanged fixation (***n*** = 26)	***P***
UDVA (mean ± SD, logMAR)	0.35 ± 0.23	0.42 ± 0.33	0.406
CDVA (mean ± SD, logMAR)	0.10 ± 0.12	0.13 ± 0.15	0.433
Astigmatism (mean ± SD, diopters)	1.54 ± 1.22	1.90 ± 1.10	0.272
Refractive Difference (mean ± SD, diopters)	0.67 ± 0.57	0.56 ± 0.64	0.618
Endothelial cell loss (mean ± SD, /mm2)	363 ± 374	226 ± 387	0.250

*CDVA* corrected distance visual acuity, *UDVA* uncorrected distance visual acuity

There was no significant difference between the two groups in vertical, horizontal or 3D tilt from the corneal topographic axis (*P* > 0.05). The differences in vertical, horizontal or 3D decentration from the corneal topographic axis between the two groups were not significant either (*P* > 0.05) (Table 3).

**Table 3:** Comparison of intraocular lens (IOL) tilt and decentration between the 2 groups at 3 months postoperatively

Parameters	Sutured fixation (***n*** = 27)	Flanged fixation (***n*** = 26)	***P***
Vertical tilt (mean ± SD, °)	5.85 ± 4.28	5.99 ± 4.65	0.904
Horizontal tilt (mean ± SD, °)	4.57 ± 2.09	4.01 ± 2.80	0.413
3D tilt (mean ± SD, °)	8.20 ± 3.76	8.22 ± 4.11	0.986
Vertical decentration (mean ± SD, mm)	0.34 ± 0.24	0.47 ± 0.30	0.099
Horizontal decentration (mean ± SD, mm)	0.43 ± 0.33	0.28 ± 0.30	0.088
3D decentration (mean ± SD, mm)	0.59 ± 0.33	0.59 ± 0.39	0.972

For vertical decentration, 70.4% in the sutured group and 88.5% in the flanged IOL fixation group decentered downward, 11.1% in the sutured group and 11.5% in the flanged IOL fixation group decentered upward. For horizontal decentration, 51.9% in the sutured group and 38.5% in the flanged IOL fixation group decentered temporally, 29.6% in the sutured group and 19.2% in the flanged IOL fixation group decentered nasally.

There was no significant difference between the two groups in all internal optical aberrations, including total internal optical aberration, internal astigmatism, total internal HOA, internal coma, horizontal and vertical internal coma, internal trefoil and internal spherical aberration (*P* > 0.05). The two groups did not differ in average height of modulation transfer function (aMTF) or Strehl ratio (SR) either (*P* > 0.05) (Table 4).

**Table 4:** Comparison of internal optical aberrations between the 2 groups at 3 months postoperatively

Optical aberrations	Sutured fixation (***n*** = 27)	Flanged fixation (***n*** = 26)	***P***
Total internal optical aberration (mean ± SD, μ)	0.596 ± 0.560	0.495 ± 0.287	0.412
Internal astigmatism (mean ± SD, μ)	0.441 ± 0.350	0.361 ± 0.240	0.333
Total internal HOA (mean ± SD, μ)	0.366 ± 0.469	0.305 ± 0.217	0.549
Internal coma (mean ± SD, μ)	0.200 ± 0.301	0.216 ± 0.185	0.825
Vertical internal coma (mean ± SD, μ)	0.169 ± 0.291	0.162 ± 0.163	0.908
Horizontal internal coma (mean ± SD, μ)	0.089 ± 0.099	0.113 ± 0.126	0.449
Internal trefoil (mean ± SD, μ)	0.184 ± 0.236	0.111 ± 0.115	0.162
Internal sphere aberration (mean ± SD, μ)	0.067 ± 0.145	0.070 ± 0.060	0.913
aMTF	0.296 ± 0.099	0.267 ± 0.069	0.230
SR	0.06748 ± 0.04409	0.04999 ± 0.02703	0.088

*HOA* higher-order aberration, *aMTF* average height of modulation transfer function, *SR* Strehl ratio

Vertical IOL decentration significantly correlated with total internal optical aberration (*r* = 0.288, *P* = 0.036), total internal HOA (*r* = 0.440, *P* = 0.001), internal coma (*r* = 0.348, *P* = 0.001), vertical internal coma (*r* = 0.388, *P* = 0.004), aMTF (*r* = − 0.364, *P* = 0.007) and SR (*r* = − 0.297, *P* = 0.031). Horizontal IOL decentrations significantly correlated with horizontal internal coma (*r* = 0.312, *P* = 0.023). IOL tilt did not correlate with any internal HOA. IOL tilt or decentration did not correlate with spherical aberration (Table 5).

**Table 5:** Correlation between IOL tilt and decentration and internal optical aberrations

IOL decentration	Optical aberrations	r^**a**^	***P***
Vertical decentration	Total internal optical aberration	0.288	0.036*
	Total internal HOA	0.440	0.001**
	Internal coma	0.348	0.001**
	Vertical internal coma	0.388	0.004**
	aMTF	−0.364	0.007**
	SR	−0.297	0.031*
Horizontal decentration	Horizontal internal coma	0.312	0.023*

*, *P <* 0.05

**, *P* < 0.01

^a^Spearman’s correlation coefficient

*HOA* higher-order aberration, *aMTF* average height of modulation transfer function, *SR* Strehl ratio

Table 6 shows the postoperative complications of the two groups. There was no difference between the two groups in early complications including hypotony (IOP ≤ 6 mmHg), vitreous hemorrhage or IOL capture. No late complication including cystoid macular edema, IOL dislocation or severe complications like endophthalmitis and retinal detachment happened during the follow-up time.

**Table 6:** Postoperative complications in sutured fixation and flanged fixation group

Complications	Sutured fixation (***n*** = 27)	Flanged fixation (***n*** = 26)	***P***
Hopotony, n(%)	2 (7.4%)	1 (7.7%)	1.000^a^
Vitreous hemorrhage, n(%)	2 (7.4%)	1 (7.7%)	1.000^a^
Iris capture of IOL, n(%)	1 (3.7%)	0 (0)	0.509^b^
Cystoid macular edema, n(%)	0 (0)	0 (0)	–
IOL dislocation, n(%)	0 (0)	0 (0)	–
Endophthalmitis, n(%)	0 (0)	0 (0)	–
Retinal detachment, n(%)	0 (0)	0 (0)	–

^a^continuous correction

^b^Fisher’s exact test

## Discussion

In eyes with insufficient capsular support, visual rehabilitation could be achieved with several IOL implantation techniques. Among them, transscleral sutured IOL fixation and intrascleral sutureless IOL fixation had been proved to be safe and effective [1, 15]. In recent years, intrascleral sutureless IOL fixation technique was favored by many surgeons because it was free of suture-related complications. Different modifications of intrascleral sutureless IOL fixation technique had been proposed since its advent in 2007 [3–8]. The flanged IOL fixation technique developed by Yamane et al. was a sutureless technique with the advantages of transconjunctival approach, short operating time and firm incarceration of the IOL haptics in the sclera [8].

The methods to measure IOL tilt and decentration had evolved in the past few decades, including Purkinje image technique, Scheimpflug photography, UBM and AS-OCT [14, 16–21]. None of the above methods could measure IOL tilt and decentration directly except for second generation AS-OCT (Casia2). Image processing software and manual measurement were necessary when using these methods. Moreover, pupillary axis was frequently used as the reference axis in the initial studies based on these methods [19]. The deviation of pupil center had a negative effect on the accuracy of measurement. Compared to the pupil center, corneal vertex was considered to be a better reference to assess IOL tilt and decentration because it was not affected by pupil shape [22]. The present study used Casia2 to measure IOL tilt and decentration. Casia2 was a second generation AS-OCT with deep scan depth, good penetration and higher resolution. It could automatically measure the tilt and decentration of IOL using the corneal topographic axis as the reference. It had been proved to have very high inter-observer and intra-observer reproducibility in the measurement of IOL tilt and decentration [22].

There was a paucity of studies comparing sutured and sutureless IOL fixation techniques [9–12]. Marianelli et al. used UBM to compare the IOL tilt between sutureless and sutured scleral fixated IOLs. No significant difference was seen in the vertical tilt and horizontal tilt [10]. Later on, two studies had compared sutured IOL fixation technique and flanged IOL fixation technique. In Sül et al.’s study using a Scheimpflug camera, flanged IOL fixation group had lower IOL tilt in both meridians and lower lenticular astigmatism than the sutured group. The two groups did not differ in IOL decentrations [11]. In another study conducted by Do et al., however, the IOL tilt and decentrations in both meridians measured by Casia2 did not differ between the two groups. The postoperative visual acuity and refractive differences did not differ either [12]. Similar to Do et al’s study, the two groups in the present study did not differ in IOL tilt and decentrations in both meridians.

Good alignment of IOL is fundamental for optimal visual function. However, a significant amount of tilt and decentration can sometimes be induced after sutured or sutureless fixation of IOLs in the absence of capsular support. IOL tilt and decentration compromise the optical performance of eyes by inducing astigmatism or HOAs [23, 24]. To our knowledge, there was previously no study comparing the HOAs between transscleral sutured IOL fixation and intrascleral sutureless IOL fixation. The present study used iTrace Visual Function Analyzer to measure the internal astigmatism and HOAs and showed that the two groups did not differ in internal astigmatism or internal HOAs. The present study also investigated the relationship between IOL tilt and decentration and internal HOAs. Among sutured and sutureless fixed IOLs, vertical decentration was found to be correlated with total internal HOA, internal coma, vertical internal coma, internal aMTF and internal SR. Horizontal decentration only correlated with horizontal internal coma. Vertical and horizontal IOL tilt did not correlate with any internal HOA. In some other studies, however, IOL tilt instead of IOL decentration was often found to be correlated with coma-like aberrations [24–26]. Same as in other studies, IOL tilt and decentration did not correlate with spherical aberration.

According to the present study, flanged IOL fixation and sutured IOL fixation had similar IOL alignment and visual outcomes. Since flanged IOL fixation could avoid suture-related complications and had shorter operating time [12], this technique could be a promising alternative to treat patients with insufficient capsular support. However, compared with sutured technique, it is more challenging to ensure good centration of IOL for flanged IOL fixation. First, the internal opening of the tunnels could not be visualized when penetrating the sclera with the needle. Second, once the haptics are externalized onto the sclera, it is almost impossible to adjust the position of the IOL. Inappropriate positioning of the insertion points and angles of the two needles may cause considerable amount of IOL decentration and tilt in flanged IOL fixation. To solve this problem, we used a corneal marker for radial keratotomy to help ensure correct positions of the insertion points of the needle. The surgeon’s experience was important to ensure correct angle of the needle and correct position of the internal opening of the tunnel. In 2019, a “needle stabilizer” was invented by Yamane et al., which was likely to be useful for surgeons with less experience in flanged IOL fixation [27]. For sutured IOL fixation technique, a corneal marker could help to reduce IOL decentration effectively.

When the results of our study are discussed, its limitations should be mentioned. First, the recruitment of the participants did not follow a random pattern, so that a confounding effect owing to a potential selection bias might have been possible. However, the two groups did not differ in baseline demographic and clinical characteristics. All of the vitrectomy was done using a 23G system and the IOL fixation techniques were not selected based on the status of the patients. Second, the follow-up time was only 12 months, so that longer-term complications could not be seen in the present study. Since breakage of 10–0 polypropylene sutures can happen 6 or more years after surgery, the advantage of flanged IOL fixation technique might have been underestimated in this study. In addition, the sample size was small. A larger sample size is needed to support our argument that the two procedures had similar visual outcomes and IOL positions. In a word, a large, randomized study with longer follow-up time is therefore necessary to confirm our results.

## Conclusions

In conclusion, sutureless flanged IOL fixation had similar IOL position and visual outcomes compared with transscleral sutured IOL fixation within 1 year postoperatively. Vertical and horizontal IOL decentrations were associated with increased internal HOAs and thus should be avoided in particular.

## Data Availability

The datasets used and/or analyzed during the current study are available from the corresponding author on reasonable request.
